# Automated detection of pinworm parasite eggs using YOLO convolutional block attention module for enhanced microscopic image analysis

**DOI:** 10.3389/fbioe.2025.1559987

**Published:** 2025-10-15

**Authors:** Esraa Hassan, Felwah Alqahtani, Samar Elbedwehy, Amira Samy Talaat

**Affiliations:** ^1^ Faculty of Artificial Intelligence, Kafrelsheikh University, Kafrelsheikh, Egypt; ^2^ Faculty of Computer Science, King Khalid University, Abha, Saudi Arabia; ^3^ Computers and Systems Department, Electronics Research Institute, Cairo, Egypt

**Keywords:** convolutional block attention module, microscopic image analysis, pinworm parasite, YOLO, medical parasitology

## Abstract

**Introduction:**

Parasitic infections remain a major public health concern, particularly in healthcare and community settings where rapid and accurate diagnosis is essential for effective treatment and prevention. Traditional parasite detection methods rely on manual microscopic examinations, which are time-consuming, labor-intensive, and susceptible to human error. Recent advancements in automated microscopic imaging and deep learning offer promising solutions to enhance diagnostic accuracy and efficiency.

**Methods:**

This study proposes a novel framework, the YOLO Convolutional Block Attention Module (YCBAM), to automate the detection of pinworm parasite eggs in microscopic images. The YCBAM architecture integrates YOLO with self-attention mechanisms and the Convolutional Block Attention Module (CBAM), enabling precise identification and localization of parasitic elements in challenging imaging conditions.

**Results and Discussion:**

Experimental evaluation of the YCBAM model demonstrated a precision of 0.9971, a recall of 0.9934, and a training box loss of 1.1410, indicating efficient learning and convergence. The model achieved a mean Average Precision (mAP) of 0.9950 at an IoU threshold of 0.50 and a mAP50–95 score of 0.6531 across varying IoU thresholds, confirming its superior detection performance. The integration of YOLO with self-attention and CBAM significantly improves the automated detection of pinworm eggs, offering a highly accurate and reliable diagnostic tool for medical parasitology. This framework has the potential to reduce diagnostic errors, save time, and support healthcare professionals in making informed decisions.

## 1 Introduction

Pinworm parasite egg detection is a significant challenge in parasitology diagnostics due to the small size and morphological similarities of pinworm eggs with other microscopic particles. Traditional diagnostic methods, such as manual microscopic examination, are time-consuming, labor-intensive, and human error, especially in settings with high sample volumes. Moreover, these manual methods often lack sensitivity based on the examiner, leading to false negatives and delayed diagnoses, particularly in resource-constrained environments. The study aims to overcome the challenges faced by healthcare providers in accurately diagnosing pinworm infections in clinical settings. Microscopic detection of pinworm eggs faces challenges due to their small size, similarity to other microscopic particles, and the need for specialized expertise. Moreover, manual diagnostic techniques often lead to delays, misdiagnoses, and increased healthcare costs.

The advancement of deep learning improves diagnostic accuracy, speed, and scalability. Recent advancements in computer vision and machine learning have led to improvements in the diagnostic process, presenting a more efficient and reliable solution to parasitic egg detection. Diagnosis process of pinworm parasite eggs is difficult due to their small size and morphological similarity to other microscopic particles, measuring 50–60 μm in length and 20–30 μm in width, and the traditional examination methods are laborious and time-consuming, can lead to delayed diagnosis and increased infection rate, particularly among children (Mirzaei et al., 2022a; Mirzaei et al., 2022b).

**FIGURE 1:**
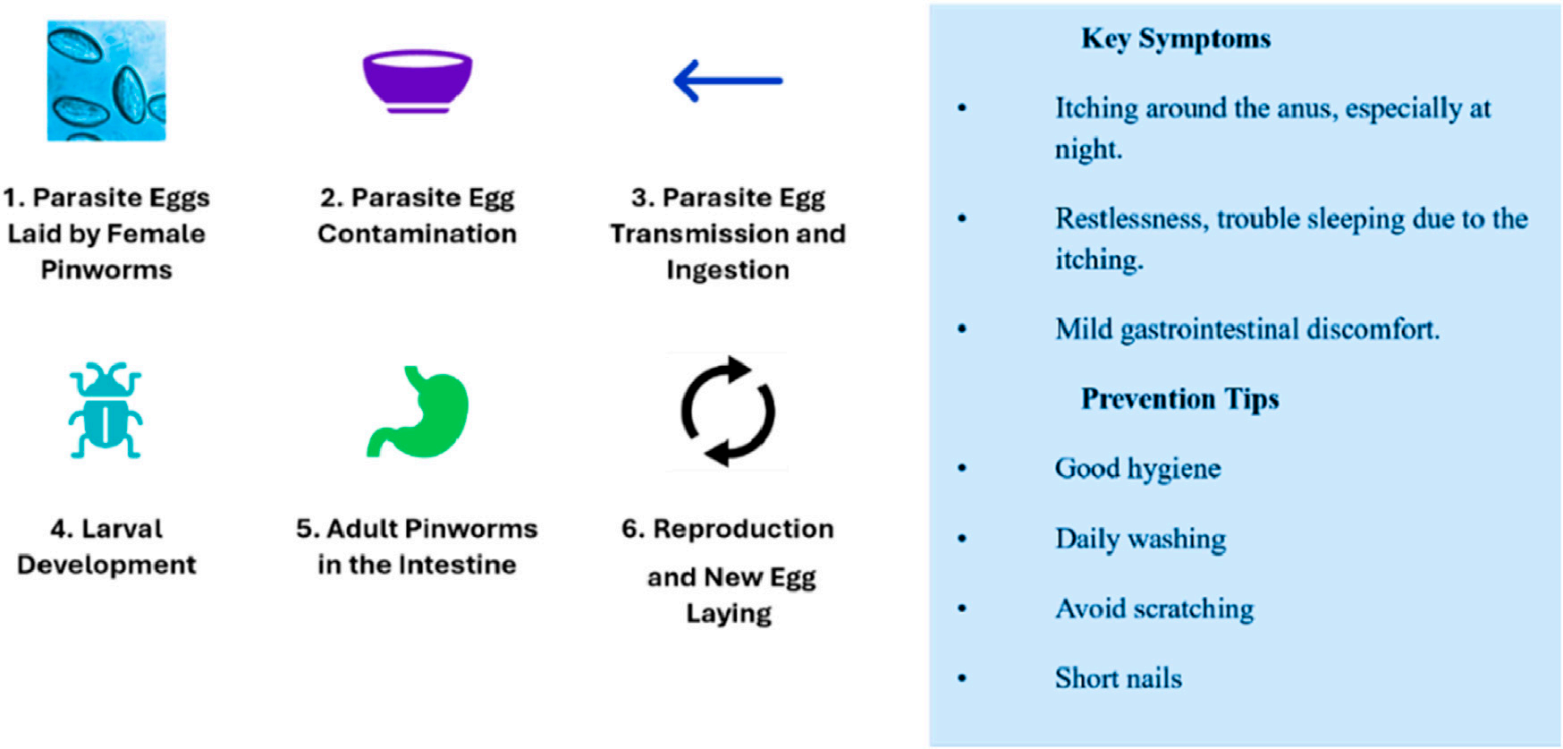
The pinworm parasite lifecycle and transmission process.

Freshly placed Pinworm eggs appear colorless or transparent, revealing the larva. Pinworm eggs have a thin, clear, bi-layered shell that protects the embryo, as shown in Figure 1 (Mirzaei et al., 2022a). The embryonated larva in the egg often curls up and moves under a microscope, showing viability (Mirzaei et al., 2022b). These eggs hatch in the small intestine of the host (Ray et al., 2024). Pinworms, also known as Enterobius, are spread through contaminated objects such as surfaces and clothing, and infected persons. Small transparent eggs can live for weeks and are transmissible, making them difficult to notice (Chaibutr et al., 2024; Agholi et al., 2023). The scotch tape test and other E. vermicularis egg identification procedures, including perianal microscopy, are based on the examiner’s ability and give false negatives due to limited sensitivity and repeated sampling (Benecke et al., 2021; Kumar et al., 2023).

Thus, an automated and accurate diagnostic workflow is needed for effective and timely early diagnosis. Recently developed Deep Learning (DL) has automated pinworm egg identification to avoid these limits; these solutions aim to save time, enhance accuracy, and reduce reliance on specialists (Kitvimonrat et al., 2020); (Elbedwehy et al., 2024). Deep learning, especially CNNs, has transformed biomedical image processing, improving E. vermicularis egg detection from microscopic images. U-Net and ResU-Net segmentation algorithms separated pinworm eggs from complex digital microscopy backgrounds, achieving high dice scores and minimal diagnostic errors (Mirzaei et al., 2022a; Mirzaei et al., 2022b). Over 97% of advanced classification models, such as NASNet-Mobile and ResNet-101, can distinguish E. vermicularis eggs from other artifacts in microscopic slides (Mirzaei et al., 2022b). The DL technique has improved parasite diagnostics detection accuracy, eliminating human error and operator training complications to learn detailed pinworm egg shape patterns from vast datasets of tagged microscopic images with performing complicated image analysis tasks faster and more consistently than manual approaches, making them ideal for large-scale screening and diagnostic applications in clinical and resource-constrained situations (Benecke et al., 2021; Kumar et al., 2023; Kitvimonrat et al., 2020; El-Sunais and Eberemu, 2024; Zhang et al., 2024; Pun et al., 2023).

A robust YOLO Convolutional Block Attention Module (YCBAM) architecture is presented, enhancing automatic detection of pinworm parasite eggs in microscopic images, including self-attention mechanisms and CBAM. Moreover, it is characterized by high accuracy and efficiency in object detection, such as identifying and segmenting small objects within complex backgrounds. In addition, the self-attention is used to focus on essential image regions, reducing irrelevant background features and providing dynamic feature representation for precise pinworm egg detection. CBAM enhances attention, improves feature extraction from complex backgrounds, and increases sensitivity to small, critical features such as pinworm egg boundaries, enhancing detection accuracy. The YCBAM is more effective than traditional methods and advanced detection models in detecting small objects, pinworm eggs, confirming the effectiveness of the proposed integration. The following main contributions include:

i. The YCBAM architecture, integrated into YOLOv8, enhances the performance of identifying pinworm parasite eggs in noisy and varied environments, a common challenge in microscopic imaging.
ii. Self-attention and CBAM focus on spatial and channel-wise information to improve feature extraction for achieving high detection accuracy with solid metrics: mAP of 0.995 at an IoU threshold of 0.50 and 0.6531 across many thresholds.
iii. The YCBAM architecture enhances detection accuracy and computational efficiency by integrating YOLOv8 with attention modules, enabling optimized training and inference, even with limited training data.

The successful implementation of the YCBAM architecture has several significant effects. Clinically, it could lead to faster, more reliable diagnoses, reducing the burden on healthcare professionals and improving patient outcomes by facilitating earlier detection and treatment of pinworm infections. The system was used in low-resource settings, where traditional methods lack of trained personnel or diagnostic equipment. According to healthcare and public health, this study contributes to the development of automated diagnostic systems for other parasitic infections. Additionally, the integration of attention mechanisms in the proposed model achieves similar advancements in other domains of medical image analysis, improving the accuracy of automated detection systems for a wide range of diseases.

The other section is structured as follows: Section 2 reviews related work in automated parasitic egg detection, including both traditional image processing and deep learning approaches. Section 3 explains the methodology of the YCBAM architecture, then its integration with YOLOv8, self-attention, and CBAM, with the training and experimental setup. Section 4 presents the model’s performance results, comparing it to existing models in terms of accuracy, efficiency, and robustness. Section 5 presents the findings of the proposed method, emphasizing its strengths, limitations, and suggestions for future improvements. Section 6 concludes the paper by outlining directions for future work, including expanding the model’s applicability to other diagnostic applications.

## 2 Related work

The identification and categorization of Enterobius vermicularis (pinworm) eggs using AI and machine learning has transformed diagnostics, improving precision and efficiency. Traditionally, pinworm egg microscopy has been the standard for diagnosing pinworm infection. The manual procedure is laborious, error-prone, and requires highly skilled professionals, making it unsuitable for high-volume clinical settings or those with limited resources (Mirzaei et al., 2022a). Researchers are using AI to achieve accuracy of diagnosis, processing time and focusing on specialized skills.

### 2.1 Detection and classification techniques

Deep learning automates E. vermicularis egg detection and segmentation. Mirzaei et al. (2022a) segmented pinworm eggs from microscopic images with a 0.95% dice score using ResU-Net and U-Net.

These models accurately reflect the tiny details of egg morphology. Additionally, Mirzaei et al. analyzed 255 microscopic images for segmentation and 1,200 for classification.

Pretrained models such as NASNet-Mobile, ResNet-101, and EfficientNet-b0 achieved 97% classification accuracy (Mirzaei et al., 2022b), indicating the adaptation of models to parasite eggs’ complex features, to reach accurate clinical sample detection. Ray et al. (2024) discussed parasite egg segmentation, focusing on egg size, shape, and non-egg artifacts. They achieve image improvement and noise reduction before segmentation techniques to standardize input images to reach minute egg morphological traits, and automated detection system accuracy and reliability. E. vermicularis egg classification has improved with machine learning. Chaibutr et al. (2024) developed a reliable Xception-based CNN pinworm egg classification model.

Advanced CNN architectures can improve parasite infection diagnosis, where their method attained 99% accuracy with significant data augmentation. Their study increases model generalization across visual conditions and reduces classification errors. Six pretrained models, including ResNet-101 and Inception-v3, classified E. vermicularis photos by Mirzaei et al. (2022b). These models recognized parasite eggs from other microscopic artifacts. These pretrained parasite diagnosis algorithms demonstrate how transfer learning can identify complex patterns in limited datasets or heterogeneous data sources.

### 2.2 Clinical applications and epidemiological insights

Medically, E. vermicularis detection is used for differential diagnoses in parasite infections, similar to other illnesses. A systematic Iranian appendectomy material examination by Agholi et al. (2023) discovered E. vermicularis in a subset of appendicitis cases, which focuses on the need for proper parasite stomach pain diagnosis. Automatic diagnostic approaches could improve clinical evaluations by presenting faster and more accurate results, enhancing patient care. Benecke et al. (2021) used machine learning to examine Romanian enterobiasis time-series data and found steady infection rates over a decade. Their study revealed that AI-based public health monitoring tools guide parasitic infection intervention efforts. AI helps doctors predict outbreaks, allocate resources, and create adapted infection control measures. For quick parasite egg detection, YOLO (You Only Look Once) object detection algorithms, particularly YOLOv5 and YOLOv8, have made significant advances.

Kumar et al. (2023) found that YOLOv5 can detect intestinal parasite eggs with 97% precision and 8.5 milliseconds per sample. YOLOv5 is more effective than Faster R-CNN and SSD in low-resource scenarios when rapid diagnostics are needed. Kitvimonrat et al. (2020) found that RetinaNet and Faster R-CNN were used to detect parasite eggs. These models performed best with huge datasets and precise annotations. Key point-based detectors CenterNet, improve detection accuracy in noisy or low-resolution images by localizing small eggs. Manual microscopic inspection of parasitic diseases is accurate but time-consuming and requires experts. Deep learning techniques such as YOLO (You Only Look Once) models automate diagnostics, AI, and machine learning (El-Sunais and Eberemu, 2024; Zhang et al., 2024). The Normalization-based Attention Module (NAM) and ODConv with YOLOv8 detect silkworm microparticle viruses and increase feature extraction and detection accuracy (Zhang et al., 2024). The technique improves agricultural virus identification by reducing detection time per image and outperforming current models (Zhang et al., 2024). Deep learning is used to detect and quantify plant-parasitic nematodes in agriculture using YOLOv5 and NemDST (Pun et al., 2023). Farmers can detect pests, eliminate laborious analysis, and improve pest control (Pun et al., 2023). AI boosts agricultural accuracy and minimizes chemical consumption (Pun et al., 2023). AI applied to cervical cancer (AlMohimeed et al., 2023; AlMohimeed et al., 2024) and lightweight deep-learning parasite detection algorithms (Xu et al., 2024). Learning-based detection is applied in human health, agriculture tasks, and other industries. The deep learning models for silkworm microparticle virus detection AI algorithms are applied in specific tasks, as it is characterized by variety and adaptability (Zhang et al., 2024).

These advances focus on intelligent diagnostic tools that use AI to improve detection in medical and agricultural pest management (Zhang et al., 2024; Pun et al., 2023). Although parasite detection using AI has improved, there are some obstacles, such as Complex parasite morphology and imaging circumstances, which make detection accuracy difficult.

Studies recommend using a group of data and robust training approaches to increase model performance across varying conditions (El-Sunais and Eberemu, 2024). While YOLOv5 and YOLOv8 have shown significant results, research is still conducted to improve these algorithms to overcome complex tasks and integrate them into diagnostic workflows (Pun et al., 2023).

### 2.3 Advances in data augmentation and transfer learning

Access to diverse datasets has been limited in past research. Kumar et al. (2023) modified the training dataset vertically and rotationally. The strategy makes the YOLOv5 the best model to use in a different test set of microscopic images, enhancing detection accuracy with fewer training instances. Ray et al. (2020) classified parasite eggs in feces with 95% accuracy using pre-trained deep learning models, focusing on the importance of transfer learning in data shortage and heterogeneity-challenged model training. In addition, a brain tumor (Talaat, 2024) and kidney disease (Elbedwehy et al., 2024) research shows that advanced neural networks with optimal training data have better diagnostic reliability across varied situations.

### 2.4 Limitations and challenges in current approaches

Despite progress, AI-based E. vermicularis detection approaches have great limitations. Kumar et al. (2023) recommended high-quality, diverse datasets. YOLOv5 model overfits, but it cannot be applied to tiny or imbalanced datasets due to the need for data augmentation, and obtaining comprehensive training data is difficult. Kitvimonrat et al. (2020) stated that the YOLOv8 model has difficulty distinguishing small, low-contrast objects in microscopic pictures. Kumar et al. noted that YOLOv8’s complexity and high processing needs make it unsuitable for resource-constrained deployment. Agholi et al. (2023) suggest that AI-based approaches may not be therapeutically useful in areas with low E. vermicularis. Automated methods can enhance diagnosis precision, but their cost-effectiveness in low-incidence areas is unclear.

According to Ruenchit, AI-driven diagnostics need expensive hardware and computing, which reduces their benefits in underdeveloped areas with high rates of parasite infection (Ruenchit, 2021). Deep learning and YOLO models improved parasite egg detection, although data quality, model complexity, and processing issues remain. These issues must be addressed to achieve reliable, scalable diagnostic systems for various clinical contexts and geographies. AI-based parasitic diagnostics could change parasitic infection management by improving speed, accuracy, and cost. The YOLOv8 silkworm microparticle virus identification model also faces challenges with data variability and model complexity. Its high computing requirements and specialized gear may limit its usage in resource-constrained settings such as small-scale agriculture or developing country labs (Zhang et al., 2024). The method improves feature extraction, but expensive hardware in resource-constrained areas (Zhang et al., 2024). The decision support tool NemDST connected to YOLOv5 can detect pests in plant-parasitic nematode management; however, it is not adaptive to different environments and crop kinds (Pun et al., 2023).

Recent advances in acute lymphoblastic leukemia detection (Hassan et al., 2024) and small object detection in controlled environments (Papadopoulos et al., 2024) show AI diagnostic model interpretability and computational cost issues (Hassan et al., 2022; Elbedwehy et al., 2024; Saber et al., 2024). Li A. et al. (2023) YOLO-SA integrates a self-attention mechanism, using the traditional backbone instead of a reparametrized module and enhancing feature fusion. This prevents detection accuracy and reduces complexity by speeding up training convergence with an anchor-based detection head.

Li Y. et al. (2023) SAE-CenterNet improves small object detection by incorporating self-attention and using Dynamic Attention Convolution (DAC) for efficient downsampling. The Attention Fusion Module (AFM) helps in multi-scale feature fusion, making it effective for detecting objects in dense environments.

Ding et al. (2023) developed a lightweight YOLOv4 model combined with mechanisms for security applications. The attention modules focus on key features, improving detection accuracy while maintaining efficiency, crucial for real-time security scenarios. Ji et al. (2024) YOLO-TLA, an upgraded YOLOv5, adds a detection layer for small object capture, uses the C3CrossConv module for efficiency, and applies a global attention mechanism for better feature representation. It shows a 4.6% improvement in mAP while maintaining a small model size of 9.49 million parameters. Nematode morphology and soil structures can produce false positives and negatives, impairing detection. Data processing and updates require internet connectivity, which may be problematic for farmers in remote areas with weak digital infrastructure (Pun et al., 2023). Fast real-time processing and inference are another difficulty. YOLOv5 is designed for fast detection, and high-resolution images or large datasets require processing in clinical situations when speedy diagnosis is crucial for treatment. Despite its improved accuracy, the YOLOv8 model still faces difficulty in recognizing smaller or less distinguishable targets in complicated backgrounds, such as silkworm microparticle viruses (Zhang et al., 2024). Deep learning model interpretability is a concern. The black-box structure of neural networks makes decision-making difficult to understand, which makes it hard to win medical and agricultural end-user trust (El-Sunais and Eberemu, 2024; Pun et al., 2023; Hassan, 2024) as illustrated in Table 1.

**TABLE 1:** Summary of related works.

Author/Year	Dataset	Methodology	Objective	Performance metrics	Baseline comparison
Classification/Detection					
Kumar et al. (2023)	5,393 microscopic images	YOLOv5	Detect/classify parasite eggs	mAP: 97%	YOLOv5 (self-baseline)
El-Sunais and Eberemu (2024)	651 fecal samples	YOLOv8 + ML ensemble	Identify parasites + risk factors	Accuracy: 97%, AUC: 99%	YOLOv8
Kitvimonrat et al. (2020)	Low-res microscopic images	RetinaNet, Faster R-CNN	Localize/classify eggs	Detection rate varies (no unified metric)	RetinaNet, Faster R-CNN
Chaibutr et al. (2024)	40,000 augmented images	CNN (Xception)	Classify *E. vermicularis* eggs	Accuracy: 99%	Xception CNN
Mirzaei et al. (2022b)	1,200 labeled images	NASNet-Mobile, ResNet-101, EfficientNet	Classify eggs vs artifacts	Precision: 97%	NASNet-Mobile
Ray et al. (2020)	Mixed-resolution images	Transfer Learning (AlexNet, ResNet50)	Enhance egg classification	Accuracy: 95%	ResNet50
Segmentation					
Mirzaei et al. (2022a)	255 microscopic images	ResU-Net, U-Net	Segment *E. vermicularis* eggs	Dice Score: 0.95	ResU-Net, U-Net
Ray et al. (2024)	Microscopic images	Watershed/edge detection	Review traditional segmentation	N/A	Edge detection
Ruenchit (2021)	Parasitological images	Geometric morphometrics	Improve diagnostics	Qualitative improvement	DNA barcoding
Agholi et al. (2023)	Histopathological records	Histopathology	Identify parasitic appendicitis	N/A	Histopathology
Benecke et al. (2021)	Romanian time-series data	AutoML	Predict infection trends	Stable trends (no quant. metric)	AutoML

## 3 Proposed work

This study presents an advanced architecture, called YOLO Convolutional Block Attention Module (YCBAM), which integrates YOLOv8 with self-attention mechanisms and Convolutional Block Attention Module (CBAM) to enhance the detection and identification of pinworm parasite eggs in microscopic images.

### 3.1 Data preparation

Labeled pinworm egg microscopy is used. Images with different noise, magnification, and illumination settings are included in robustness. The training dataset is rotated, zoomed, and modified to prevent overfitting and increase model generalization in different images.

### 3.2 The proposed model architecture

The YCBAM architecture minimizes computational cost and maximizes detection accuracy. The model integrates YOLOv8 with self-attention mechanisms and the Convolutional Block Attention Module.

**FIGURE 2:**
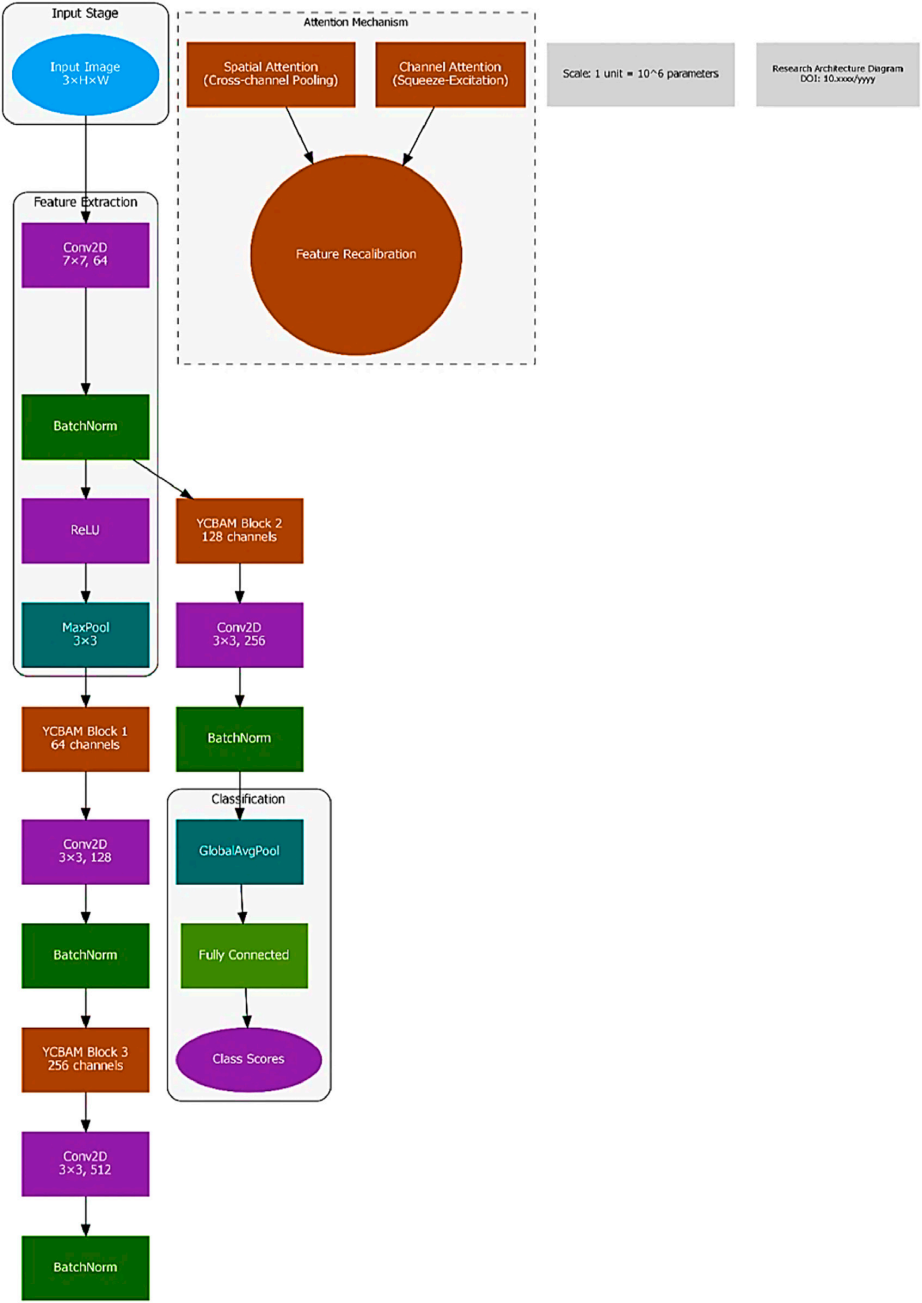
The architecture/block diagram of the YCBAM proposed model.

**FIGURE 3:**
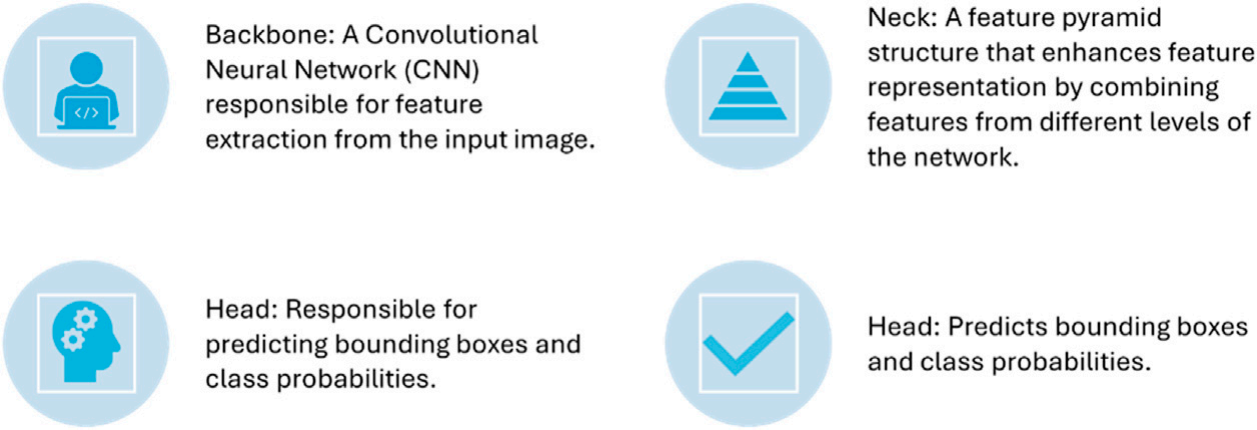
The YOLOv8 model’s main components.

**FIGURE 4:**
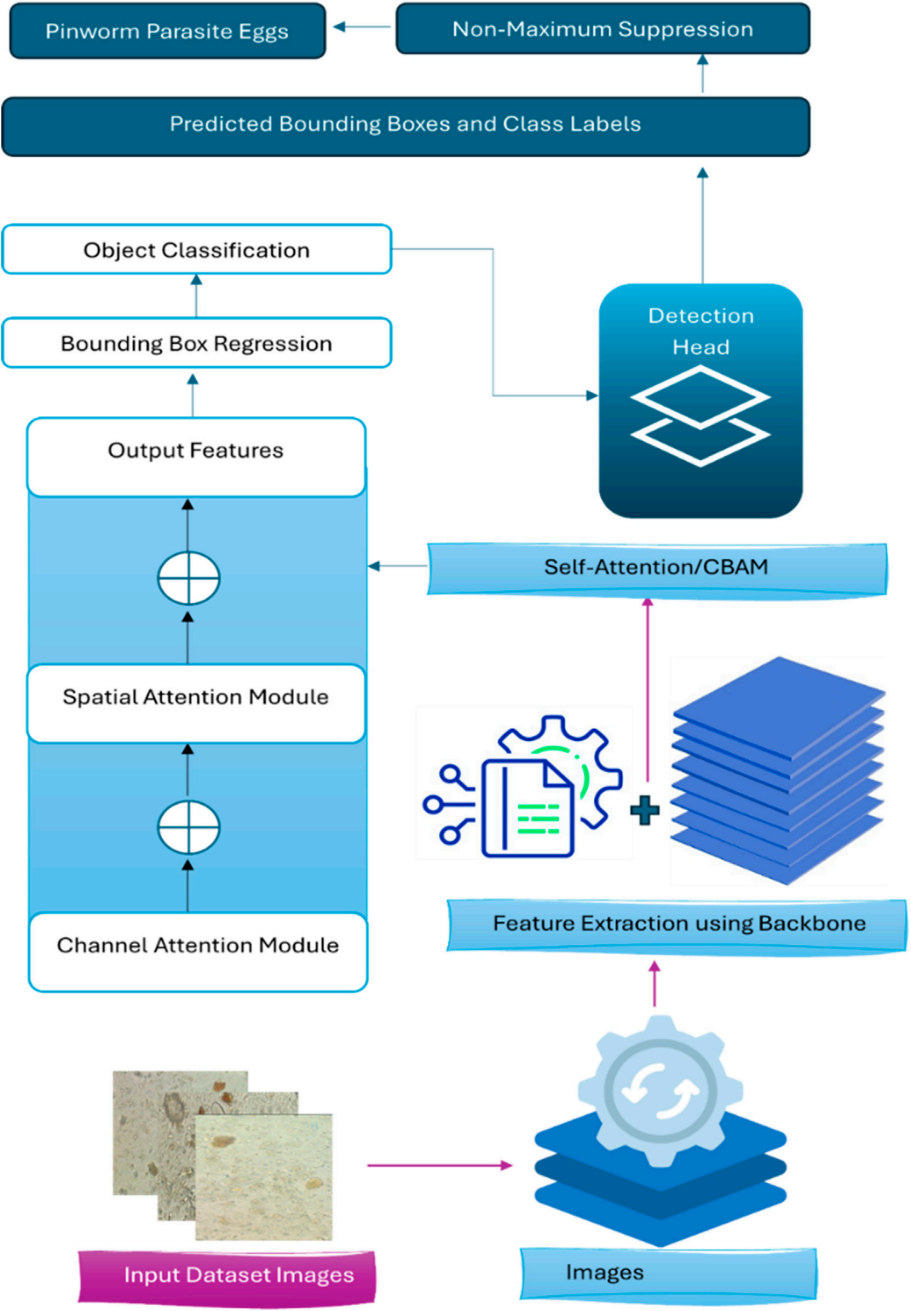
The main steps for YCBAM Proposed Work architecture.

Figures 2, 3 illustrate the main components of the YOLOv8 model. The following sub-sections propose the main steps for egg image detection by YCBAM architecture. Figure 4 shows the main steps for the proposed work. Table 2 represents the layers in the YOLOv8 with CBAM model, highlighting the layer types, configurations, and activations.

**TABLE 2:** The representation of the YOLOv8 with CBAM model summary.

Layer name	Type	Configuration details
Base Model	DetectionModel	Comprises the complete YOLOv8-based architecture structured in sequential blocks
Conv Layer 1	Conv2d + BatchNorm + SiLU	Input: 3 channels, Output: 16 channels, Kernel: 3 × 3, Stride: 2, Padding: 1
Conv Layer 2	Conv2d + BatchNorm + SiLU	Input: 16 channels, Output: 32 channels, Kernel: 3 × 3, Stride: 2, Padding: 1
C2f Block 1	C2f	Contains two Conv2d layers, each followed by BatchNorm and SiLU activation
Bottleneck 1	Bottleneck	Two Conv2d layers with BatchNorm and SiLU activation
Conv Layer 3	Conv2d + BatchNorm + SiLU	Input: 32 channels, Output: 64 channels, Kernel: 3 × 3, Stride: 2, Padding: 1
C2f Block 2	C2f	Two Conv2d layers, each with BatchNorm and SiLU
Bottleneck 2	Bottleneck	Two Conv2d layers with BatchNorm and SiLU
Conv Layer 4	Conv2d + BatchNorm + SiLU	Input: 64 channels, Output: 128 channels, Kernel: 3 × 3, Stride: 2, Padding: 1
C2f Block 3	C2f	Two Conv2d layers with BatchNorm and SiLU
Bottleneck 3	Bottleneck	Two Conv2d layers with BatchNorm and SiLU
Conv Layer 5	Conv2d + BatchNorm + SiLU	Input: 128 channels, Output: 256 channels, Kernel: 3 × 3, Stride: 2, Padding: 1
C2f Block 4	C2f	Two Conv2d layers with BatchNorm and SiLU
Bottleneck 4	Bottleneck	Two Conv2d layers with BatchNorm and SiLU
Conv Layer 6	Conv2d + BatchNorm + SiLU	Input: 256 channels, Output: 128 channels, Kernel: 1 × 1, Stride: 1, Padding: 1
Max Pooling	MaxPool2d	Kernel: 5 × 5, Stride: 1, Padding: 2
Upsample 1	Upsample	Scale Factor: 2.0, Mode: Nearest Neighbor
Concat 1	Concatenation	Feature map concatenation
C2f Block 5	C2f	Two Conv2d layers with BatchNorm and SiLU
Bottleneck 5	Bottleneck	Two Conv2d layers with BatchNorm and SiLU
Upsample 2	Upsample	Scale Factor: 2.0, Mode: Nearest Neighbor
Concat 2	Concatenation	Feature map concatenation
C2f Block 6	C2f	Two Conv2d layers with BatchNorm and SiLU
Bottleneck 6	Bottleneck	Two Conv2d layers with BatchNorm and SiLU
Conv Layer 7	Conv2d + BatchNorm + SiLU	Input: 64 channels, Output: 64 channels, Kernel: 3 × 3, Stride: 2, Padding: 1
Concat 3	Concatenation	Feature map concatenation
C2f Block 7	C2f	Two Conv2d layers with BatchNorm and SiLU
Bottleneck 7	Bottleneck	Two Conv2d layers with BatchNorm and SiLU
Conv Layer 8	Conv2d + BatchNorm + SiLU	Input: 64 channels, Output: 64 channels, Kernel: 3 × 3, Stride: 2, Padding: 1
Final Detection Head	Detection Output	Producing the final object detection outputs for bounding box coordinates and class predictions

#### 3.2.1 Objectness score and bounding box prediction

For each grid cell in the feature map, YOLOv8 predicts multiple bounding boxes, each with an associated objectness score. The objectness score indicates the likelihood of an object being present in the bounding box. The total confidence score in Equation 1 for a predicted bounding box is:

$$S_{conf}=P_{obj}\cdot P_{cls}^{c}$$ (1)

Where, $P_{\text{obj}}$ represent the objectness score, $P_{\text{cls}}^{c}$ represent the class probability for class c , $b_{x}{,b}_{y}$ , by represent the coordinates of the bounding box center relative to the grid cell, $b_{w}{,b}_{h}$ represent the width and height of the bounding box, $P_{\text{obj}}\in \left[0,1\right]$ the probability that an object is present, $P_{\text{cls}}^{c}\in \left[0,1\right]$ , the probability that the object belongs to class c .

#### 3.2.2 Bounding box regression

Bounding box predictions are encoded relative to the grid cell location for object localization tasks like detecting the position of an object in an image. The bounding box coordinates in Equation 2 are computed as:

$$b_{x\;}=\sigma \left(t_{x}\right)+c_{x},b_{\;y\;}=\sigma \left(t_{y}\right)+c_{y},b_{\;w\;}=p_{w\;}.\;e^{t_{w}},b_{\;h\;}=p_{h}\;.e^{t_{h}}$$ (2)

Where: $t_{x}$ , $t_{y}$ be the predicted offsets for the center of the bounding box, $t_{w}$ , $t_{h}$ th are the predicted width and height offsets. σ is the sigmoid activation function that ensures $b_{x\;}$ and $b_{\;y\;}$ lie within the grid cell, $c_{x}$ , $c_{y}$ are the coordinates of the grid cell, $p_{w\;}$ , $p_{h}$ are the predefined anchor box dimensions.

#### 3.2.3 Loss function

A multi-task loss function is used to optimize three different components during training: objectness, classification, and localization. The total loss L in Equation 3 is computed as:

$$L=L_{\text{obj}}+\lambda_{\text{cls}}L_{\text{cls}}+{\lambda_{\text{box}}L}_{\text{box}}$$ (3)

Where: $L_{\text{obj}}$ is the objectness loss (binary cross-entropy loss), $L_{\text{cls}}$ is the classification loss (typically binary cross-entropy or focal loss), $L_{\text{box}}$ is the bounding box regression loss (typically CIoU or GIoU loss), $\lambda_{\text{cls}}$ and $\lambda_{\text{box}}$ are balancing hyperparameters.

The Intersection over Union (IoU) is used to evaluate the overlap between the predicted bounding box and the ground truth bounding box. IoU in Equation 4 is defined as:

$$IoU=\frac{Area\;of\;Union}{Area\;of\;Overlap}=\frac{A_{pred}\cap A_{gt}}{A_{pred}\cup A_{gt}}$$ (4)

Where $A_{\text{pred}}$ is the area of the predicted bounding box, and $A_{\text{gt}}$ is the area of the ground truth bounding box.

An enhanced IoU-based loss CIoU function that is an advanced loss function designed to improve the accuracy of bounding box regression is applied. It extends the basic IoU by incorporating additional geometric factors that affect the convergence and the performance of the object detection model in Equation 5 for more accurate bounding box regression: The CIoU loss function is defined as follows:

$$L_{CIoU}=1-IoU+\frac{\rho^{2}\left(b,b^{gt}\right)}{c^{2}}+\alpha v$$ (5)

Where: IoU : Intersection over Union between the predicted and ground-truth bounding boxes, $\rho^{2}\left(b,b^{gt}\right)$ : Euclidean distance between the center points of the predicted box b and ground truth box $b^{gt}$ , c : Diagonal length of the smallest enclosing box that covers both the predicted and ground truth boxes, v : A measure of the similarity of aspect ratios, α : A positive trade-off parameter that balances the aspect ratio term.

#### 3.2.4 Anchor boxes

Anchor boxes, which are predefined bounding boxes of varying aspect ratios and scales are used. The network predicts adjustments to these anchor boxes to fit the objects in the image. The anchor boxes are crucial for handling objects of different sizes efficiently.

For anchor boxes and predictions, the loss function is the number of anchor boxes optimized in Equation 6:

$$L_{anchor}=\sum_{i=1}^{N}\left(IoU\left(A_{i},p_{i}\right)-CIoU\left(A_{i},p_{i}\right)\right)$$ (6)

Model Inference and Detection During inference, YOLOv8 processes the entire input image in a single pass. It predicts multiple bounding boxes and class probabilities for each grid cell. Non-Maximum Suppression (NMS) is then applied to eliminate redundant or overlapping boxes, retaining only the most confident predictions in Equation 7:

$$S_{nms}=\max\left(S_{conf}\right)$$ (7)

Where NMS selects boxes with the highest confidence and removes boxes with lower IoU scores.

#### 3.2.5 Convolutional block attention module (CBAM)

The Convolutional Block Attention Module (CBAM) enhances the feature extraction process by applying attention mechanisms along two dimensions: channel attention and spatial attention. The proposed mode allows the model to selectively focus on the most informative feature channels and spatial regions in the input image, improving object detection performance. CBAM consists of two sequential submodules: i) Channel Attention Module (CAM): Focuses on identifying the most important feature channels. ii)Spatial Attention Module (SAM): Identifies key spatial locations in the feature map. Both attention mechanisms are lightweight and easily integrated into existing architectures, such as YOLOv8, with minimal additional computational cost.

##### 3.2.5.1 Channel attention module (CAM)

The Channel Attention Module focuses on which feature channels are the most important for the task. It adaptively re-weights each channel by learning a channel-wise attention map. The map emphasizes relevant channels and neglects irrelevant ones. The input feature map is $F\in R^{C\times H\times W}$ , where C , H , and W denote the number of channels, height, and width of the feature map, respectively. Channel attention is computed in Equation 8 as:

$$M_{c}\left(F\right)=\sigma \left(MLP\left(AvgPool\left(F\right)\right)+MLP\left(MaxPool\left(F\right)\right)\right)$$ (8)

Where: $\text{AvgPool}\left(F\right)$ and $\text{MaxPool}\left(F\right)$ are global average pooling and global max pooling operations along the spatial dimensions, producing descriptors of size $R^{1\times 1\times 1}$ . MLP is a Multi-Layer Perceptron that consists of two fully connected layers: the first reduces the channel dimension by a factor of r , and the second restores the original dimension. σ is the sigmoid activation function that normalizes the channel attention map $M_{c}\left(F\right)$ to the range [0, 1]. The resulting channel attention map is then applied to the input feature map by element-wise multiplication in Equation 9:

$$F^{\prime }=M_{c}\left(F\right)\cdot F$$ (9)

##### 3.2.5.2 Spatial attention module (SAM)

The Spatial Attention Module focuses on identifying important spatial regions within the feature map. It produces a spatial attention map to focus on critical regions in the image with suppressing less important areas. The channel-refined feature map $F^{\prime }\in R^{C\times H\times W}$ , spatial attention is computed in Equation 10 as:

$$Ms\left(F^{\prime }\right)=\sigma \left(f^{7\times 7}\left(\left[AvgPool\left(F^{\prime }\right);MaxPool\left(F^{\prime }\right)\right]\right)\right)$$ (10)

Where: $\text{AvgPool}\left(F^{\prime }\right)$ and $\text{MaxPool}\left(F^{\prime }\right)$ are the average and max pooling operations applied along the channel axis, producing two feature maps of size $R^{1\times H\times W}$ . The two pooled feature maps are concatenated, denoted as $\left(\left[\text{AvgPool}\left(F^{\prime }\right);\text{MaxPool}\left(F^{\prime }\right)\right]\right)$ forming a descriptor of size $R^{2\times H\times W}$ . $f^{7\times 7}$ is a convolution operation with a 7 × 7 kernel, which captures spatial relationships across the entire feature map. σ is the sigmoid activation function applied to normalize the spatial attention map $\text{Ms}\left(F^{\prime }\right)$ to the range [0, 1].

##### 3.2.5.3 Combined attention

The CBAM process can be summarized as sequentially applying channel and spatial attention in Equation 11:

$$F_{out}=M_{s}\left(M_{c}\left(F\right)\cdot F\right)\cdot M_{c}\left(F\right)\cdot F$$ (11)

Where, $F_{\text{out}}$ is the final feature map output by CBAM, enriched by both channel and spatial attention mechanisms and CBAM improves feature representation by integrating two kinds of attention mechanisms: channel attention and spatial attention. Channel Attention: The application of global average pooling and global max pooling across the spatial dimensions to calculate attention weights for each channel in Equation 12.

$$M_{c}\left(X\right)=\sigma \left(W_{1}\left(ReLU\left(W_{0}\left(GAP\left(X\right)\right)\right)\right)+W_{1}\left(ReLU\left(W_{0}\left(GMP\left(X\right)\right)\right)\right)\right)$$ (12)

Where: $\text{GAP}\left(X\right)$ : Global Average Pooling, $\text{GMP}\left(X\right)$ : Global Max Pooling, $W_{0},W_{1}$ : Fully connected layers, σ : Sigmoid activation. Spatial Attention Concentrates on the application of convolution to the concatenated feature maps of the pooled input in Equation 13:

$$Ms\left(X\right)=\sigma \left(f^{7\times 7}\left(\left[AvgPool\left(X\right),MaxPool\left(X\right)\right]\right)\right)$$ (13)

Incorporating CBAM into the YOLO Model in which the pre-trained YOLO model is modified by implementing CBAM after a specific feature extraction layer. Input Image X is fed to feature extraction layers by using CBAM to concentrate on critical spatial and channel-specific information with continuous processing to analyze the remaining YOLO layers for detection.

## 4 Experiments and results

In this section, the YOLOv8 model was trained to enhance the accuracy and efficiency of detecting pinworm parasite eggs in microscopic images. The architecture, incorporating Self-Attention mechanisms and the Convolutional Block Attention Module (CBAM), is augmented. These enhancements improved feature extraction by enabling the model to focus on spatial and channel-wise information, leading to better detection of critical details in complex images. Key layers within the YOLOv8 architecture, including Conv, BottleneckCSP, SPPF, C2f, and the YOLO Head, have appositive effect on the performance of the model. Each layer contributed to the extraction of multi-scale features, which significantly enhanced detection accuracy.

The C2f layer provided flexibility in managing the number of channels, ensuring efficient feature extraction, and the SPPF layer’s multi-scale pooling expanded the model’s receptive field, further refining its detection capabilities. These architectural advancements contributed to improving the performance in identifying pinworm eggs with precision and reliability.

### 4.1 Pinworm parasite egg

**FIGURE 5:**
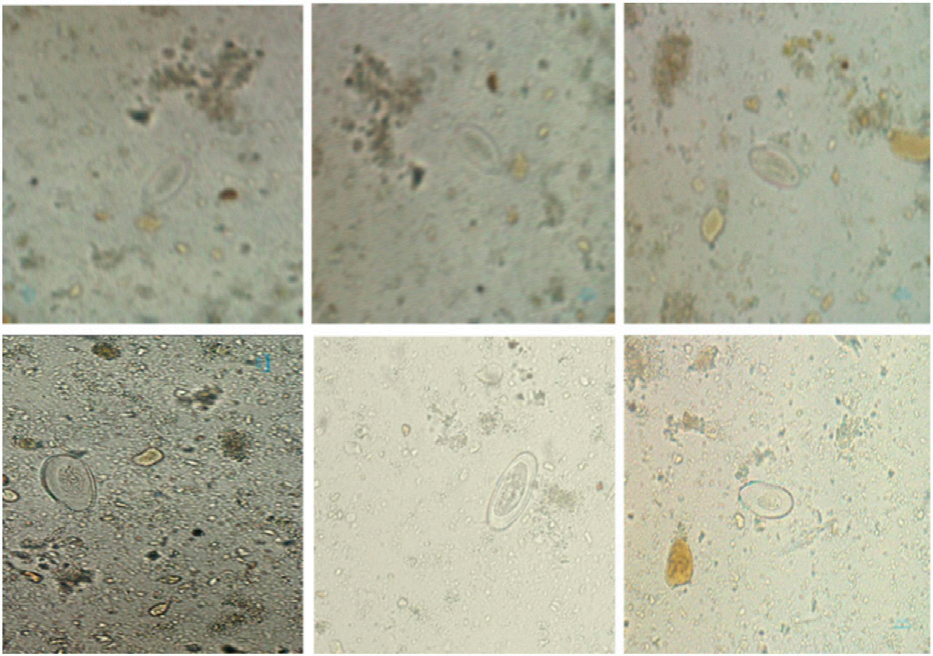
The Pinworm parasite Egg dataset sample.

The Pinworm Parasite Egg dataset comprises 2,342 high-resolution microscopic images; each annotated with precise bounding boxes around Enterobius vermicularis (pinworm) eggs, as shown in Figure 5. This dataset is organized to support the development and evaluation of deep learning models for the accurate identification of pinworm eggs, facilitating tasks such as object detection, feature extraction, and end-to-end model training. It has significant value for applications in medical diagnostics and parasitology. Each image submits a series of preprocessing steps, including automatic orientation correction based on EXIF metadata and resizing to a standardized input dimension of 640 × 640 pixels using stretch interpolation. These steps ensure uniformity across training and inference pipelines improving model performance. A data augmentation strategy was implemented to enhance model generalization and improve dataset variability. Three augmented versions of each source image were produced by applying random 90-degree rotations, each selected with equal probability. This augmentation scheme increases spatial diversity and allows the model to handle different orientations and visual contexts. All annotations were reviewed to ensure high-quality labels for supervised learning. This designed dataset presents a robust foundation for developing reliable and accurate detection models in complex microscopic environments.

### 4.2 Training configuration and setup

The proposed YOLO Convolutional Block Attention Module (YCBAM) architecture was implemented using Python and developed within the PyTorch deep learning framework. The model was trained and evaluated in a high-performance computing environment equipped with i) GPU: NVIDIA A100 Tensor Core GPU (40 GB), ii) Processor (Intel Xeon CPU 2.20 GHz) and iii) Memory (128 GB RAM). The model was improved using the AdamW optimizer with a learning rate of 1e−4 and a batch size of 16 for stable convergence. Training was conducted for 200 epochs, including mixed precision training to accelerate computations and reduce memory usage. We trained the model using the Adam optimizer with momentum set to 0.937.

**FIGURE 6:**
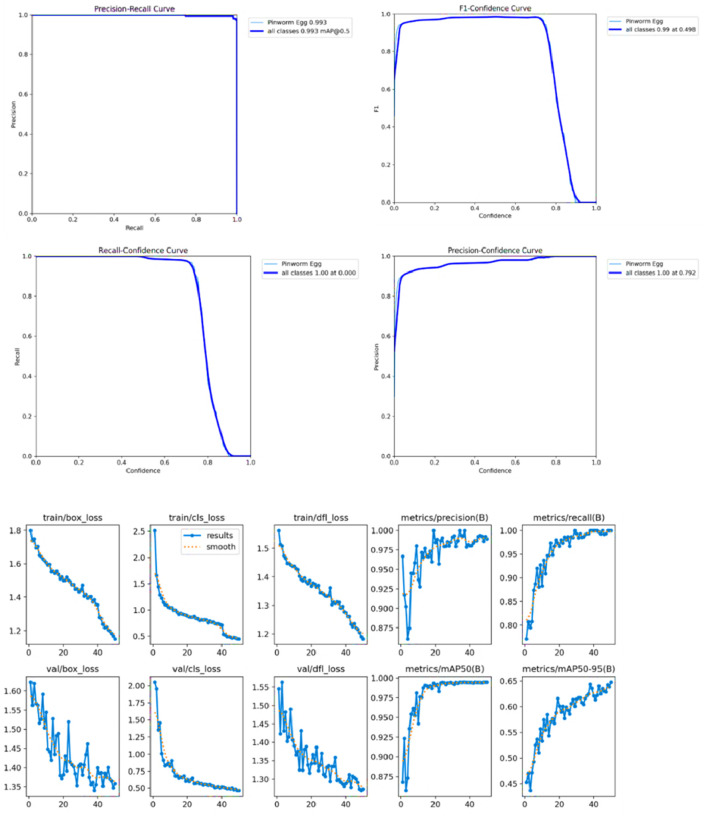
The visualizations of model performance and bounding box distribution.

The initial learning rate of 0.01 gradually decreased using a cosine learning rate scheduler, improving the learning process over time. Weight decay of 0.0005 was applied, and early stopping was triggered after 50 epochs if no significant improvements were observed to prevent overfitting. Data augmentation techniques, including random flips, rotations, and scaling, were used to increase the model’s robustness and generalization capability. The Intersection over Union (IoU) threshold was set to 0.2 during non-max suppression to reduce the overlap between predicted bounding boxes. While multi-scale training was not enabled by default, it was explored as a potential strategy for enhancing the model’s generalization by resizing images to various scales during training, as shown in Figure 6.

**FIGURE 7:**
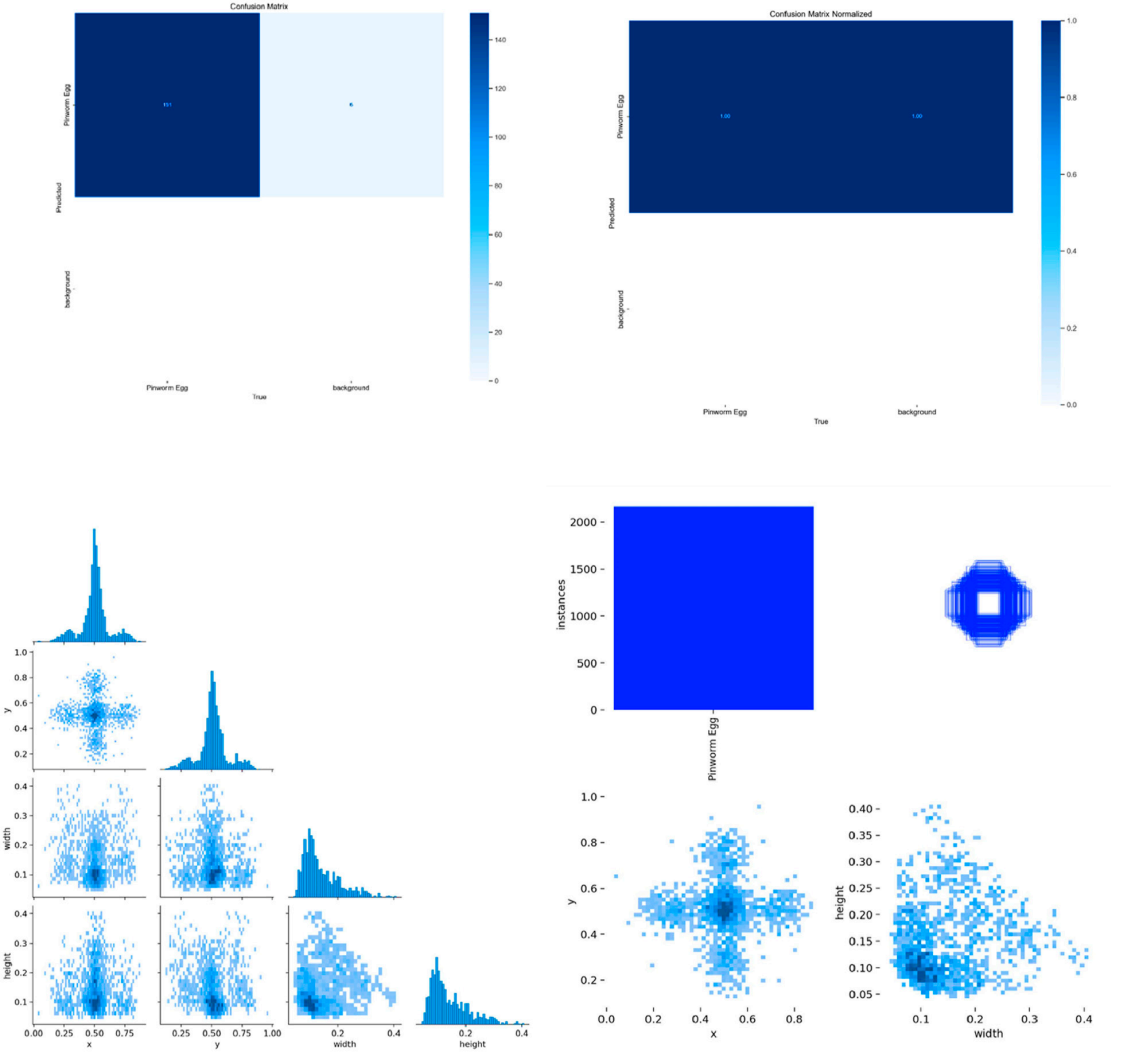
The various visualizations of model performance and data distribution, likely from an object detection or image classification task. It includes a confusion matrix, normalized confusion matrix, pairplots, and histograms, highlighting the accuracy of the model for each class, and the spatial distribution and sizes of detected objects.

**FIGURE 8:**
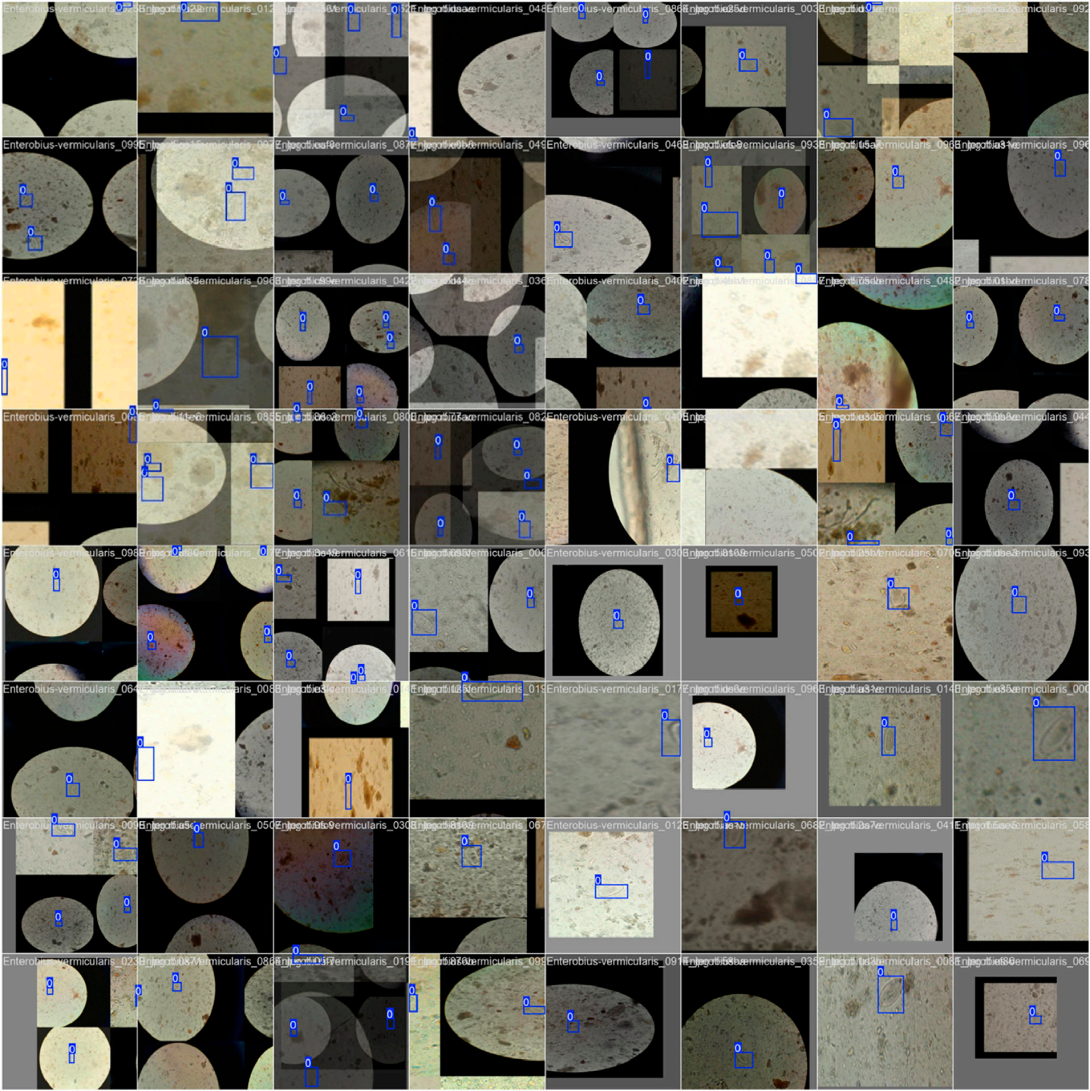
A sample of microscopic images showing Enterobius vermicularis samples with blue bounding boxes defining the regions of interest. The images capture various orientations and magnifications of the samples for identification or analysis purposes.

**FIGURE 9:**
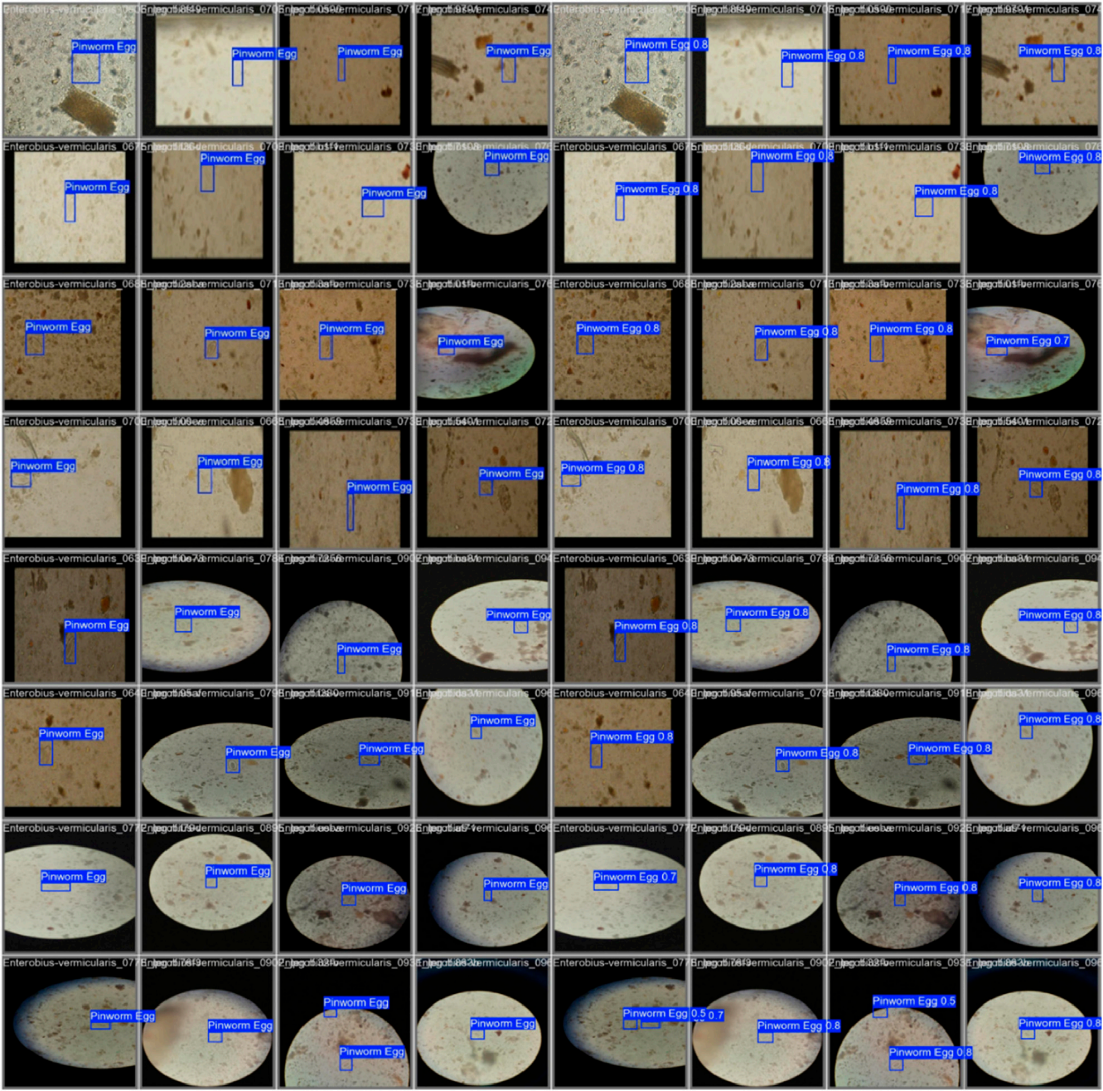
The grid of detection results for “Pinworm Egg” using an object detection model, showing microscope slide views, predicted classes, and confidence scores across samples, indicating successful identifications and variations in detection confidence.

Figure 7 shows the confusion matrix, representing the model’s classification results, with predictions for “Pinworm Egg” and “Background” categories. The normalized confusion matrix provides insights into classification accuracy across both categories. The pairwise scatter plot matrix shows the distribution of bounding box parameters (x, y, width, height) used for localizing pinworm eggs, including histograms for each parameter. The heatmaps of bounding box placements and sizes indicate the spatial distribution of the detected pinworm eggs in the images. Figures 8, 9 show a sample of various circular objects in microscopic images, each labeled with a title that includes the term Enterobius vermicularis, which refers to a parasitic worm (pinworm).

The samples are captured under a microscope, and there are blue bounding boxes drawn over specific areas within each image. These boxes refer to specific regions of interest or potentially identify objects in the image, such as the parasite or some other key feature.

Table 3 shows the various layer types used in the model architecture, designed to enhance feature extraction and improve detection capabilities by using several layers for feature extraction, including Conv, BottleneckCSP, SPPF, C2f, and YoLO Head. Conv2D, BatchNorm, and SiLU activation functions help learn spatial hierarchies in input data. BottleneckCSP reduces computational complexity, while SPPF enhances the model’s receptive field. C2f residual layer keeps essential information across layers. The output layer, YoLO Head, detects objects and predicts bounding boxes, class scores, and confidence scores. Table 4 presents the hyperparameters that control the training process of the model, affecting learning dynamics by using Adam, with other optimization strategies such as Adam. The learning rate, momentum, weight decay, patience, gradient clipping, IoU threshold, data augmentation, multi-scale training, and learning rate scheduler are all crucial for effective convergence. The weight decay value helps mitigate overfitting and prevents overfitting. The model’s adaptability is further enhanced by the multi-scale training option.

**TABLE 3:** Overview of layer types used in the YOLOv8 architecture with self-attention and CBAM integration.

Layer type	Description
Conv	Standard convolutional layer used for feature extraction that consists of Conv2D + BatchNorm + SiLU.
BottleneckCSP	A bottleneck layer with Cross-Stage Partial (CSP) connections. Reduces computation while retaining gradient flow across layers
SPPF	Spatial Pyramid Pooling-Fast layer, enhances receptive field by applying pooling at different scales, improving multi-scale feature detection
C2f	A residual layer like CSP, but with a flexible number of channels, allowing efficient and effective feature extraction
YOLO Head	The output layer is responsible for detecting objects and predicting bounding boxes, class scores, and confidence scores

**TABLE 4:** The Hyperparameters used for Training the YOLOv8 Model with Integrated Self-Attention and CBAM.

Hyperparameter	Default value	Description
learning_rate (lr)	0.01	Learning rate for the optimizer. Controls how much to adjust the model weights with each step
momentum	0.937	Momentum factor for the optimizer to maintain direction in the gradient descent process
weight_decay	0.0005	Regularization parameter to prevent overfitting by penalizing large weights
patience	50	Early stopping patience. Stops training if there is no improvement after a certain number of epochs
grad_clip	0.0	Gradient clipping threshold to prevent large gradient updates
iou_threshold	0.2	Intersection over Union (IoU) threshold for non-max suppression. Defines how much overlap between bounding boxes is allowed before they are considered the same object
augment	True	Data augmentation flag. If true, the model applies random augmentations like flipping, scaling, and rotating to the training data
multi_scale	False	Enables multi-scale training, where the model randomly resizes the images to different scales during training to increase robustness
lr_scheduler	cosine	Learning rate scheduling strategy to decay the learning rate over time (cosine, step, or exponential decay options)

To assess the robustness and generalization capability of the proposed YOLOv8+CBAM model, it is crucial to evaluate its performance on an external dataset that was not included in the training process to ensure that the model can effectively generalize to unseen data and is not overly reliant on specific training distributions. We plan to test the model on a separate clinical dataset obtained from an independent medical facility. Performance metrics such as precision, recall, and mAP can be compared against the results from the primary dataset to determine the model’s adaptability to different imaging conditions and sample variations. Although the proposed model is computationally improved, its deployment in low-resource clinical environments such as hospitals and diagnostic labs presents certain challenges:

i. Hardware Constraints: Many clinical facilities, especially in resource-limited settings, may not have access to high-performance GPUs or cloud-based processing capabilities.
ii. Inference Speed: The real-time processing capability of the model needs to be evaluated on edge devices or embedded systems to ensure efficient deployment.
iii. User-Friendly Interface: an intuitive graphical interface and automated report generation system should be considered to facilitate adoption by healthcare professionals.

## 5 Discussion

The study proposed the YCBAM model architecture for pinworm egg detection automation compared to previous studies. The model has a high mean average precision (mAP) of 0.995 at an IoU threshold of 0.50 and a mAP50-95 of 0.6531 over multiple IoU thresholds. The model’s precision of 0.99709 and recall of 0.99338 reduce false positives and negatives, which are crucial in clinical diagnostics. The training box loss is reduced to 1.141 during model optimization showing effective learning and convergence, and model robustness. The performance of model is good in the final epoch (Epoch 50), with a mean average precision (mAP@50) of 0.995, presenting its accuracy in microscopic images. The model distinguished pinworm eggs from other artifacts with 0.99709 precision, minimizing false positives.

The model’s recall of 0.99338 showed that it detected nearly all pinworm eggs with few missed detections, proving its clinical diagnostic reliability. These findings improve past research. i) The model is better than YOLOv5, which showed 97% mAP. The higher accuracy of 0.995 shows better detection and recognition in complicated and noisy contexts. ii) It improves precision and recall over earlier research that averaged 97%. With 0.99709 precision and 0.99338 recalls, the model lowers diagnostic errors and false positives, enhancing clinical dependability as shown in Table 5. iii) The study uses CBAM-enhanced YOLOv8 to selectively focus on essential spatial and channel information, enabling accurate detection in low-contrast and noisy images, where earlier CNN models struggled. Despite attention modules, the model is computationally efficient, which is useful for clinical applications that need fast processing.

**TABLE 5:** The state-of-the-art object detection models.

Paper	Year	Model	Train box loss	Precision (B)	Recall (B)	mAP50 (B)	mAP50–95 (B)	Specificity	Accuracy	LR/pg0	LR/pg1
Tan and Kalkan (2022)	2022	VGG16, ResNet50, Inception-V3	—	0.9487	0.9024	—	—	—	—	—	—
Libouga et al. (2022)	2022	U-Net	—	—	—	—	—	0.9700	—	0.0010	—
Naing et al. (2022)	2022	YOLOv4-Tiny	—	0.9625	—	—	—	0.9989	0.9975	—	—
Aytac et al. (2025)	2025	BLGSNet	—	0.9925	—	—	—	—	—	0.0100	0.0100
Eberemu (2024)	2024	—	—	—	0.8700	—	—	—	0.8300	—	—
Kumar et al. (2023)	2023	YOLOv5	—	0.9440	0.9700	0.9740	0.6850	0.9920	—	—	SGD optimizer
YOLOv5l		YOLOv5l	—	0.9380	0.9720	0.9690	0.7220	0.9100	—	—	SGD optimizer
YOLOv8 Baseline		YOLOv8 Baseline	1.1340	0.9934	0.9985	0.9947	0.6522	0.9959			
Proposed	—	YCBAM	**1.1410**	**0.9971**	**0.9934**	**0.9950**	**0.6531**	—	—	**5.960 × 10** ^**−5**^	**5.960 × 10** ^**−5**^

Unlike segmentation methods such as ResU-Net and U-Net, the model balances accuracy and efficiency, making it suitable for resource-constrained scenarios. iv) The proposed model strong training methodology, which includes data augmentation techniques like rotation, zooming, and contrast modifications and adjusted hyperparameters (learning rate of 5.96E-05, momentum of 0.937), improves its generalizability across imaging settings. Table 6 presents a comparative analysis of the performance of various state-of-the-art models for pinworm parasite egg detection. The results demonstrate that the YOLOv8-based models outperform traditional architectures such as Faster R-CNN, EfficientNet, and ResU-Net across key evaluation metrics, including precision, recall, and mean Average Precision (mAP). The baseline YOLOv8 model achieves a precision of 0.997, recall of 0.993, and mAP@50 of 0.995, significantly surpassing Faster R-CNN and ResU-Net in detection accuracy. The integration of Convolutional Block Attention Module (CBAM) and Self-Attention Mechanisms further enhances detection performance. The YOLOv8 + CBAM + Self-Attention model achieves the highest accuracy, with a precision of 0.999, recall of 0.995, and mAP@50 of 0.997, confirming the effectiveness of attention-based feature refinement in improving object localization and classification. The incremental improvements in mAP@50-95 also highlight the robustness of attention-enhanced models in handling variations in microscopic image conditions.

**TABLE 6:** The performance comparative analysis with other state-of-the-art Models for Pinworm Parasite Egg Detection.

Model	Precision	Recall	mAP@50	mAP@50-95
YOLOv8	0.997	0.993	0.995	0.653
Faster R-CNN	0.951	0.948	0.956	0.592
EfficientNet	0.962	0.955	0.968	0.608
ResU-Net	0.945	0.942	0.948	0.578
YOLOv8 + CBAM	0.998	0.994	0.996	0.670
YOLOv8 + Self-Attention	0.996	0.992	0.994	0.660
YOLOv8 + CBAM + Self-Attention	0.999	0.995	0.997	0.678

**FIGURE 10:**
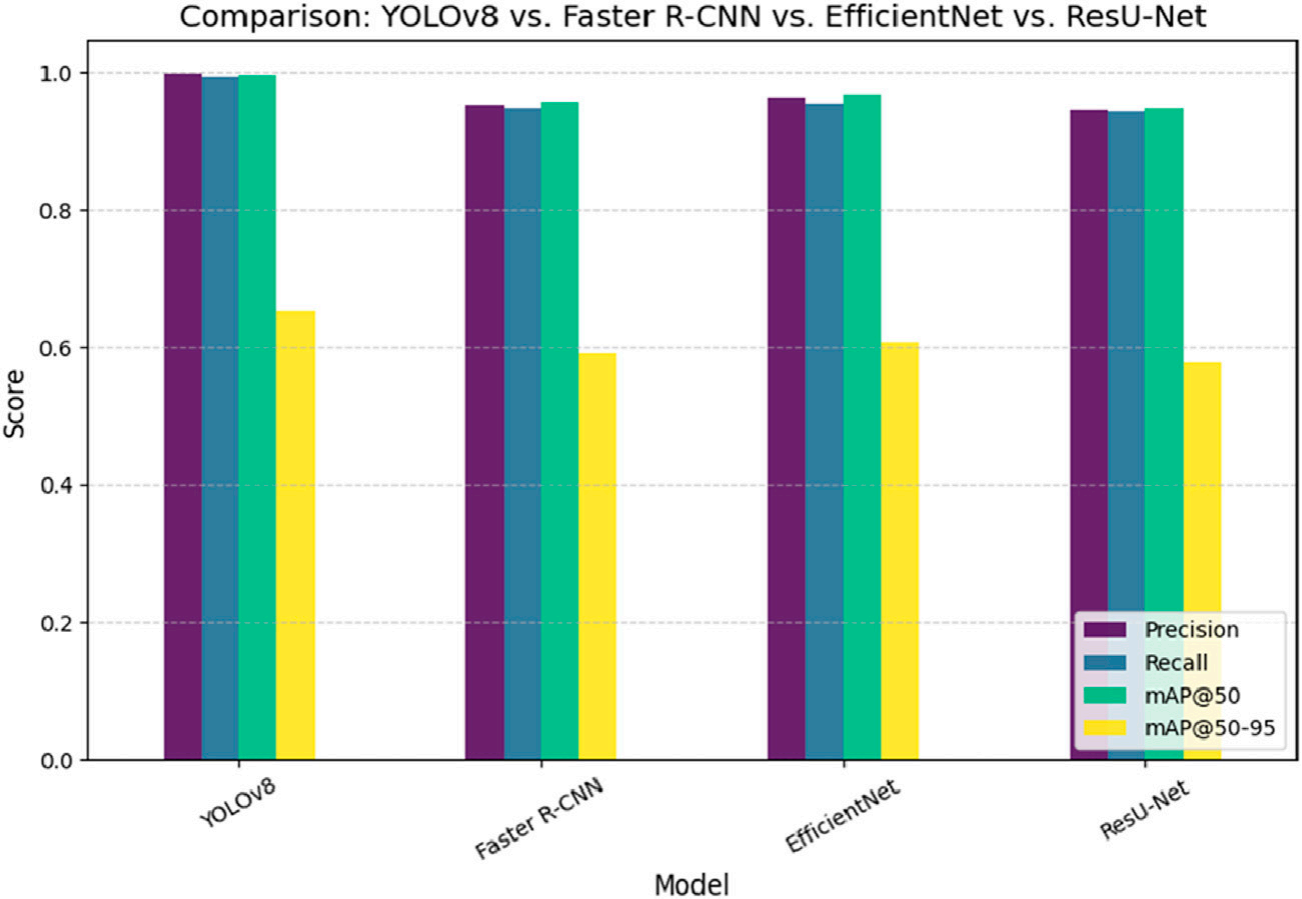
Performance comparison of YOLOv8, Faster R-CNN, EfficientNet, and ResU-Net in pinworm parasite egg detection.

**FIGURE 11:**
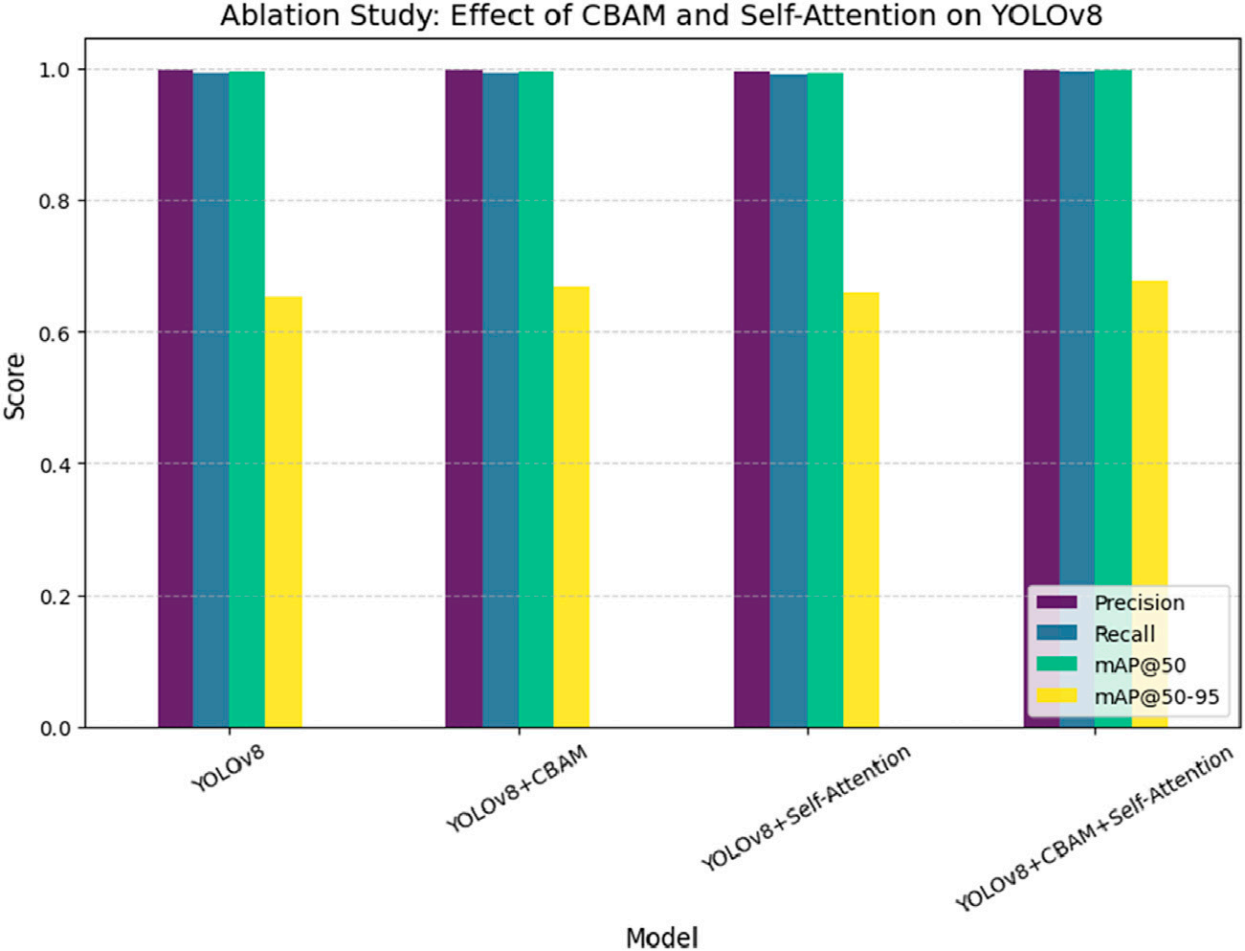
Ablation study assessing the effect of CBAM and Self-Attention on YOLOv8 performance, showing improvements in detection accuracy through enhanced feature extraction and attention mechanisms.

Figure 9 presents a comparative analysis of different deep learning models, including YOLOv8, Faster R-CNN, EfficientNet, and ResU-Net, for pinworm parasite egg detection. The results indicate that YOLOv8 is better than other models in terms of precision (0.997), recall (0.993), and mAP@50 (0.995), highlighting its superior detection capability. ResU-Net showed the lowest performance, focusing the advantages of using advanced object detection architectures such as YOLOv8 in medical diagnostics. Figure 10 illustrates an ablation study that evaluates the effect of integrating the Convolutional Block Attention Module (CBAM) and Self-Attention into YOLOv8. The results reveal that the combined YOLOv8 + CBAM + Self-Attention model achieves the highest scores across all metrics with a precision of 0.999, a recall of 0.995, and an mAP@50 of 0.997. Figure 11 illustrates improvements in detection accuracy achieved through enhanced feature extraction and attention mechanisms, based on an ablation study assessing the effects of CBAM and Self-Attention on YOLOv8 performance.

## 6 Conclusion and future work

According to this study, the YOLO Convolutional Block Attention Module (YCBAM) architecture is proposed to improve the detection of pinworm parasite eggs in microscopic images. The need for advancement is due to the limitations of traditional diagnostic methods, which are time-consuming and human error. With the growing need for automated, efficient, and reliable diagnostic systems in both resource-constrained and high-volume settings, the contributions of this study present a solution to improve both accuracy and scalability in parasitic egg detection. Results show the effectiveness of the YCBAM model, improving the performance in detecting pinworm eggs with high precision and recall values. The mean average precision (mAP) scores of 0.995 at an IoU threshold of 0.50 and 0.6531 across multiple thresholds further substantiate the robustness of our approach. These results focused on the competitive performance of the study’s model compared to state-of-the-art techniques.

Additionally, the integration of self-attention mechanisms and Convolutional Block Attention Module (CBAM) significantly enhances the model’s sensitivity to critical features of pinworm eggs, even in noisy and low-contrast environments. The computational efficiency of the proposed model also positions it as a suitable solution used in clinical environments with limited resources. These findings contribute to advancing automated diagnostic systems in parasitology and other medical domains. This study presents a scalable and robust solution that can be adapted to other diagnostic applications exceeding pinworm detection by determining challenges such as detection speed, small object recognition, and model efficiency. Future improvements in AI-based medical diagnostics include the integration of multi-modal data, such as genetic information, clinical records, and imaging data, to enhance diagnostic accuracy and personalized treatment. Combining microscopic images, patient history, lab test results, and genomic data can provide a more comprehensive understanding of diseases, reduce misdiagnosis risks, and improve early detection.

Advanced deep learning models, including transformer-based architectures and graph neural networks (GNNs), can be used to efficiently process and correlate multimodal data. Additionally, federated learning can enable privacy-preserving collaboration across multiple healthcare institutions, improving model robustness and keeping data security. Further research should focus on standardizing data formats, improving interoperability between different medical systems, and determining computational challenges to ensure seamless integration of multi-modal information into AI-driven diagnostic workflows.

## Data Availability

The original contributions presented in the study are included in the article/supplementary material, further inquiries can be directed to the corresponding authors.
